# The results of PHQ-9 screening of latvian general population in 2019-2020

**DOI:** 10.1192/j.eurpsy.2021.867

**Published:** 2021-08-13

**Authors:** V.V. Vinogradova, A. Kivite-Urtane, J. Vrublevska, E. Rancans

**Affiliations:** 1 Department Of Psychiatry And Narcology, Riga Stradins University, Riga, Latvia; 2 Department Of Public Health And Epidemiology, Riga Stradins University, Riga, Latvia

**Keywords:** PHQ-9, general_population, Screening, depressive_episode

## Abstract

**Introduction:**

Under-diagnosis of depression is a concerning problem for Latvia. According to our previous research at least 115 000 new cases have to be diagnosed each year, but the data of National Health Service show that most of the cases remain undiagnosed and untreated.

**Objectives:**

To determine the point prevalence of depressive episode and associated factors in Latvian general population.

**Methods:**

Computer assisted face-to-face interviews were carried out between November 2019 and March 2020 to gather information on a representative sample of the Latvian adult population (n=2687). The study sample was selected using a stratified random sampling method. The participants were interviewed using the Patient Health Questionnaire-9; a score of ≥10 was defined as indicating the presence of a clinically relevant depressive symptoms. Multinomial logistic regression was applied.

**Results:**

There were 1238 males (46.1%) and 1449 females (53.9%) recruited. Mean age of respondents was 49.9 (SD 18.2). The point prevalence of depressive episode in general population was 6.5% with statistically significant difference between genders: 4.8% in men and 7.7% in women (p=0.02). Lower level of education (p<0.001) and unemployment (p=0.01) were statistically significant associated factors for depressive episode among women. The odds of having depressive episode were higher in male urban dwellers (p=0.03) (but not in the capital city) and in man, who live separately, are divorced or widowed (p=0.004).

**Conclusions:**

Females, especially unemployed women and those with unfinished education, are at particular significant risk of depression, which should be adressed in developing prevention strategy and screening programmes of depression.

